# Unveiling an Unusual Organism: Catheter-Related Bloodstream Infection Due to Acinetobacter lwoffii

**DOI:** 10.7759/cureus.80318

**Published:** 2025-03-09

**Authors:** Arundhati Diwan, Sujata Khatal, Rohan Kelkar, Komal Singh, Mahendra Deoker

**Affiliations:** 1 Internal Medicine, Bharati Vidyapeeth (Deemed to be University) Medical College, Pune, IND; 2 Infectious Diseases, Bharati Vidyapeeth (Deemed to be University) Medical College, Pune, IND

**Keywords:** acinetobacter species, catheter-related bloodstream infections, hemodialysis related, nosocomial and opportunistic infections, prevention of infections

## Abstract

Catheter-related bloodstream infections (CRBSIs) are serious complications in patients requiring long-term intravascular catheterization, particularly in immunocompromised individuals. *Acinetobacter lwoffii*, a low-virulence, non-fermentative gram-negative bacillus, is an uncommon cause of CRBSI. We report the case of a 43-year-old female patient on long-term hemodialysis who developed *A. lwoffii*-associated CRBSI. This case highlights the diagnostic challenges, management strategies, and importance of early intervention in infections caused by *A. lwoffii.*

## Introduction

Vascular catheters are increasingly being used in nosocomial settings. While they are used to administer intravenous fluids, blood products, antibiotics, and drugs, and as an access for hemodialysis, one of the leading complications associated with such devices is infection. Catheter-related bloodstream infection (CRBSI) is the most common potentially fatal complication associated with central venous catheterization [1]. Skin commensals such as staphylococci and fungi are most frequently associated with these infections [2].

The genus *Acinetobacter* consists of aerobic, gram-negative coccobacilli found in water and soil. While *Acinetobacter baumannii* is known to cause nosocomial infections in immunocompromised patients [3], the clinical relevance of other *Acinetobacter* species is not very clear. *Acinetobacter lwoffii* is typically considered a commensal organism of human skin, perineum, and oropharynx [4] with low pathogenic potential. However, it can act as an opportunistic pathogen in immunocompromised individuals in nosocomial settings [2,4-6], especially those with indwelling vascular catheters leading to CRBSI. The rarity of *A. lwoffii*-associated CRBSI poses diagnostic and therapeutic challenges. Here, we present a case of CRBSI caused by *A. lwoffii* in a patient undergoing long-term hemodialysis.

## Case presentation

A 43-year-old female patient with a medical history of end-stage renal disease (ESRD) requiring maintenance hemodialysis for the past one year, type 2 diabetes mellitus (T2DM) for 15 years, and hypertension (HTN) for 10 years presented with a 10-day history of fever and chills occurring during dialysis sessions. On examination, she had a tunneled double-lumen hemodialysis catheter inserted in her right internal jugular vein. Also, she was febrile (100.1°F) with a pulse rate of 104 bpm and elevated blood pressure (160/90 mmHg). There was localized erythema and swelling around the catheter insertion site, but local hygiene was unremarkable.

Laboratory test results are shown in Table 1. At admission, the patient had a significantly elevated white blood cell count of 22,000/mm³ (reference range: 4,000-11,000/µL) and procalcitonin level > 100 ng/mL (reference range: <0.05 ng/mL), strongly suggesting the presence of systemic sepsis, likely bacterial. Her blood urea nitrogen value was 53.2 mg/dL (reference range: 4.67-21 mg/dL), and her creatinine value was 10.02 mg/dL (reference range: 0.6-1.2 mg/dL), consistent with end-stage renal disease. A transthoracic echocardiogram was performed to screen for infective endocarditis, which was normal.

**Table 1:** Laboratory test results BUN: blood urea nitrogen

Laboratory test	Day 1	Day 6	Day 10
Total leucocyte count (cells/µL)	22,000	4,100	4,600
Serum procalcitonin (ng/mL)	>100	34.5	-
Serum creatinine (mg/dL)	10.02	4.68	4.9
Serum BUN (mg/dL)	53.2	21	24

The in situ dialysis catheter was removed, and its tip was sent for culture along with blood from a peripheral vein. A 24 cm (11.5 Fr) non-tunneled double-lumen hemodialysis catheter was inserted in the right femoral vein in the groin region to facilitate dialysis. Empirical antibiotic therapy with intravenous meropenem was initiated, given its broad-spectrum coverage. Blood culture results from both the catheter tip and peripheral vein confirmed the isolation of *A. lwoffii *with a differential time to positivity being six hours, establishing the diagnosis of catheter-related bloodstream infection (CRBSI). Antibiotic susceptibility testing demonstrated that the isolate was sensitive to several antimicrobial agents, including ceftriaxone, cefepime, cefoperazone/sulbactam, gentamicin, trimethoprim/sulfamethoxazole, and ciprofloxacin. Accordingly, the patient was started on intravenous ceftriaxone (1 g every 12 hours). Supportive measures included the administration of antipyretics and insulin therapy to optimize her glycemic control.

The patient's fever resolved within 48 hours of initiating targeted antibiotic therapy with ceftriaxone, and follow-up blood cultures were sterile, confirming the resolution of the infection. A tunneled hemodialysis catheter was inserted in the left internal jugular vein after 10 days. At the four-week follow-up, no recurrence of infection was noted, and the patient remained stable without further complications.

## Discussion

*Acinetobacter lwoffii* is considered a low-virulence skin and environmental commensal bacterium, although few cases of bacteremia have been reported [2,4,7,8]. It is often considered to be a contaminant when isolated from blood culture. This can lead to a delayed or missed diagnosis, especially in immunocompromised patients in whom they can cause severe infections that can lead to sepsis, multiorgan dysfunction, or prolonged bacteremia if not promptly treated.

Clinical correlation, repeated positive cultures and an assessment of patient risk factors are necessary for an accurate diagnosis. In our case, the resolution of symptoms after catheter removal and appropriate antibiotic therapy strongly supported *A. lwoffii* as the causative pathogen.

Unlike the more virulent *A. baumannii*, *A. lwoffii* CRBSI is associated with a better prognosis when promptly treated. This involves catheter removal and targeted antimicrobial therapy [2]. In our case, *A. lwoffii* demonstrated susceptibility to ceftriaxone, cefepime, cefoperazone/sulbactam, gentamicin, trimethoprim/sulfamethoxazole, and ciprofloxacin. While historically, it was susceptible to multiple antibiotics, reports of resistance to carbapenems and cephalosporins have emerged, necessitating antimicrobial stewardship [9]. If resistance patterns are not considered, empirical antibiotic therapy may fail, which could lead to treatment delays and poor patient outcomes.

*Acinetobacter lwoffii* and other nosocomial pathogens spread in hospital settings via contaminated medical equipment, healthcare workers' hands, or ambient surfaces. Prevention of dialysis-related bloodstream infections requires a multipronged strategy consisting of core interventions, healthcare workers, and patient training and surveillance. Core interventions include proper hand hygiene, catheter hub disinfection, chlorhexidine skin antisepsis, antimicrobial lock use, and prompt catheter removal in favor of arteriovenous fistulas or grafts. Healthcare staff should be trained about catheter access care and aseptic technique and evaluated periodically. Patients should be educated about catheter care, hand hygiene, risks related to catheter use, and recognizing signs of infection. Dialysis facilities should conduct regular surveillance, perform audits, and provide feedback on infection rates [10].

This case underscores the importance of distinguishing between true infections and contamination, performing comprehensive antibiotic susceptibility testing to guide therapy, and ensuring early removal of the infected catheter, which is critical for infection resolution. Preventive strategies, such as hand hygiene, adherence to aseptic techniques during catheter insertion and maintenance, and removing unnecessary devices are essential in reducing the incidence of CRBSIs [11,12].

## Conclusions

This case highlights the significance of considering *A. lwoffii* as a potential cause of CRBSI, especially in immunocompromised patients. While often dismissed as a contaminant, its pathogenic potential should not be overlooked. Early recognition, targeted therapy, and strict catheter care protocols are essential to achieve optimal patient outcomes. There is a need to continuously study evolving resistance patterns, which can help improve clinical management strategies.
